# Immunoglobulin G4-related Disease: An Overview

**DOI:** 10.31662/jmaj.2018-0017

**Published:** 2019-01-21

**Authors:** Shigeyuki Kawa

**Affiliations:** 1 Department of Internal Medicine, Matsumoto Dental University, Shiojiri, Japan

**Keywords:** IgG4, IgG4-related disease, autoimmune pancreatitis, Mikulicz’s disease

## Abstract

Immunoglobulin G4-related disease (IgG4-RD) is a recently established systemic disease that is characteristically associated with elevated serum immunoglobulin G4 (IgG4) levels and believed to be caused by autoimmune mechanisms. The clinical features of IgG4-RD include (i) systemic distribution, (ii) imaging findings of swelling, nodules, and/or wall thickening, (iii) high serum IgG4 levels, (iv) abundant IgG4-bearing plasma cell infiltration and fibrosis in affected organs, (v) a favorable response to corticosteroid therapy, and (vi) coexistence with other IgG4-RD manifestations simultaneously or in a metachronous fashion. The concept of IgG4-RD was established based on the culmination of specific discoveries. Specifically, a close association between autoimmune pancreatitis (AIP) and high serum IgG4 levels, massive IgG4-bearing plasma cell infiltration in pancreatic tissues affected by AIP, and systemic other organ involvements in AIP with similar IgG4-bearing plasma cell features opened the gateway from AIP to IgG4-RD. The systemic distribution of IgG4-RD seems to be capable of affecting every organ, causing well-established members including AIP, lacrimal and salivary gland lesions such as Mikulicz’s disease, respiratory diseases, sclerosing cholangitis, kidney diseases, and retroperitoneal fibrosis. IgG4-RD has been diagnosed worldwide, and international collaboration efforts on the disease have led to consensus publications on its nomenclature, pathology findings, and management approach. The algorithms developed for the comprehensive diagnostic criteria for IgG4-RD have remarkably increased detection sensitivity. Oral glucocorticoids are the first-line agents for remission induction, and certain patients with high disease activity may benefit from maintenance therapy afterwards. Originally, IgG4-RD had been considered reversible and to have a good prognosis; however, long-term afflictions sometimes result in transition to advanced-stage conditions with dysfunction and/or complicating malignancy. The immunological abnormalities in IgG4-RD have been reported in both innate and adaptive immune systems; however, it remains unclear whether IgG4 has a pathogenic role or a protective one in disease onset and progression.

## Concept of IgG4-related Disease

Immunoglobulin G4-related disease (IgG4-RD) is a recently established systemic disease that is characteristically associated with IgG4 ^(1)^. IgG4-RD can be caused by autoimmune mechanisms, with following clinical features: (i) systemic distribution, (ii) imaging findings of swelling, nodules, and/or wall thickening, (iii) high serum IgG4 levels ^(2)^, (iv) abundant IgG4-bearing plasma cell infiltration and fibrosis in affected organs ^(3)^, (v) a favorable response to corticosteroid therapy, and (vi) coexistence with other IgG4-RDs simultaneously or in a metachronous fashion.

**Figure 1.**
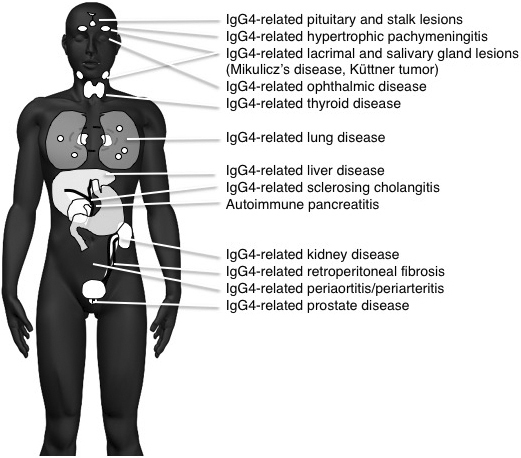
Systemic distribution of IgG4-RD.

The systemic distribution of IgG4-RD seems to be capable of affecting every organ, causing well-established members including autoimmune pancreatitis (AIP), Mikulicz’ disease, respiratory disease, sclerosing cholangitis, kidney disease, and retroperitoneal fibrosis (Figure 1). With new members getting added to IgG4-RD, the imaging findings of swelling, nodules, and/or wall thickening, which may mimic those of existing conditions in each organ, need differentiation, especially from malignant conditions; many IgG4-RD cases are still misdiagnosed and treated erroneously. Since IgG4-RD responds well to corticosteroid therapy, the correct diagnosis based on its recognition as a systemic disease is important. High serum IgG4 levels and abundant IgG4-bearing plasma cell infiltration in affected organs are the most important characteristic features of IgG4-RD and are the major indicators in the diagnostic process. However, because these findings are similar for other inflammatory or malignant conditions, their presence does not necessarily exclude other disorders. The coexistence of IgG4-RDs simultaneously or in a metachronous fashion also aids in the correct diagnosis of IgG4-RD, with many IgG4-RDs representing a highly active disease state.

## How Was the Concept of IgG4-RD Established?

The establishment of IgG4-RD was based on the culmination of specific discoveries: (1) the proposal of AIP presumably caused by autoimmune mechanisms ^(4)^; (2) a close association between AIP and high serum IgG4 levels ^(2)^; (3) abundant IgG4-bearing plasma cell infiltration in affected pancreatic tissues of AIP ^(3)^; (4) systemic other organ involvements (OOIs) in AIP also responding to corticosteroid therapy ^(5), (6)^; (5) OOIs also exhibiting abundant IgG4-bearing plasma cell infiltration ^(3)^; and (6) these findings combining to establish the concept of a systemic disease consisting of AIP and other systemic OOIs, i.e., IgG4-RD ^(7)^. These sequential discoveries led the way right from AIP to IgG4-RD.

### (1) Proposal of AIP

Pancreatitis is mainly caused by alcohol abuse. In 1961, Sarles described that pancreatitis is presumably caused by autoimmune mechanisms that showed hyperglobulinemia and marked lymphocytic infiltration ^(8)^. In 1978, Nakano reported a case of Sjögren’s syndrome (Mikulicz’s disease) with a pancreatic tumor successfully treated with corticosteroids ^(9)^. In 1991, Kawaguchi described a new pancreatic disease with lymphoplasmacytic infiltration, storiform fibrosis, and obstructive phlebitis as lymphoplasmacytic sclerosing pancreatitis ^(10)^. The following year, Toki reported a new pancreatic disease with peculiar imaging findings of diffuse irregular narrowing of the main pancreatic duct ^(11)^. In 1995, Yoshida proposed the concept of AIP based on the clinical findings of these newly reported cases, which also included hypergammaglobulinemia, autoantibody positivity, lymphoplasmacytic infiltration in the pancreas, and a favorable response to corticosteroid treatment ^(4)^.

### (2) Association between AIP and high serum IgG4 levels

**Figure 2.**
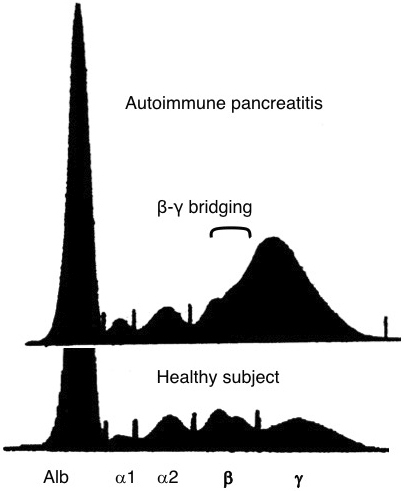
Gel electrophoresis of serum in AIP reveals β-γ globulin bridging. (From Tan to Sui 22;603–608:2001 in a Japanese publication. Reprinted with permission from Igakutosho-Shuppan, Ltd.)

**Figure 3.**
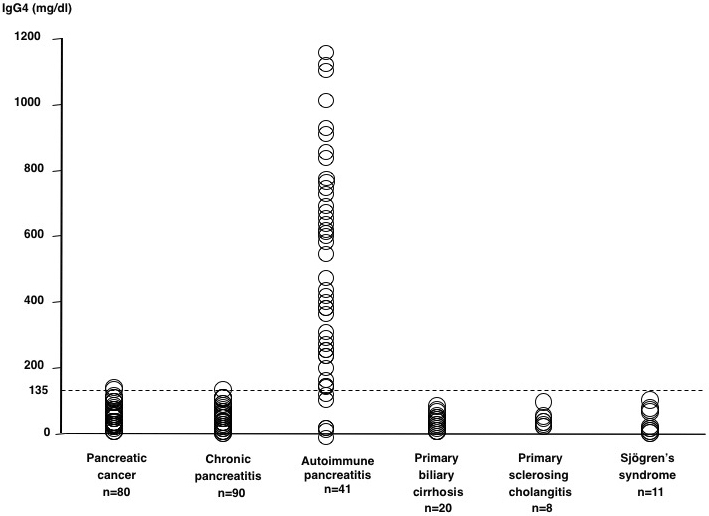
Scattergram of serum IgG4 values in various conditions, including AIP. (From Ref. 2, Copyright (C) 2001 Massachusetts Medical Society. Reprinted with permission.)

In 2001, we reported a close relationship between AIP and high serum IgG4 levels ^(2)^. Protein electrophoresis of sera of patients with AIP showed β-γ bridging, which was a polyclonal band in the rapidly migrating fraction of γ-globulins (Figure 2). This abnormal band was confirmed by immunoelectrophoresis to be caused by elevated serum IgG4 levels. We observed elevated IgG4 levels in 90% of patients with AIP but scarcely in other conditions (Figure 3), which nominated IgG4 as a sensitive and specific marker for AIP.

### (3) Abundant IgG4-bearing plasma cell infiltration in affected pancreatic tissues of AIP

**Figure 4.**
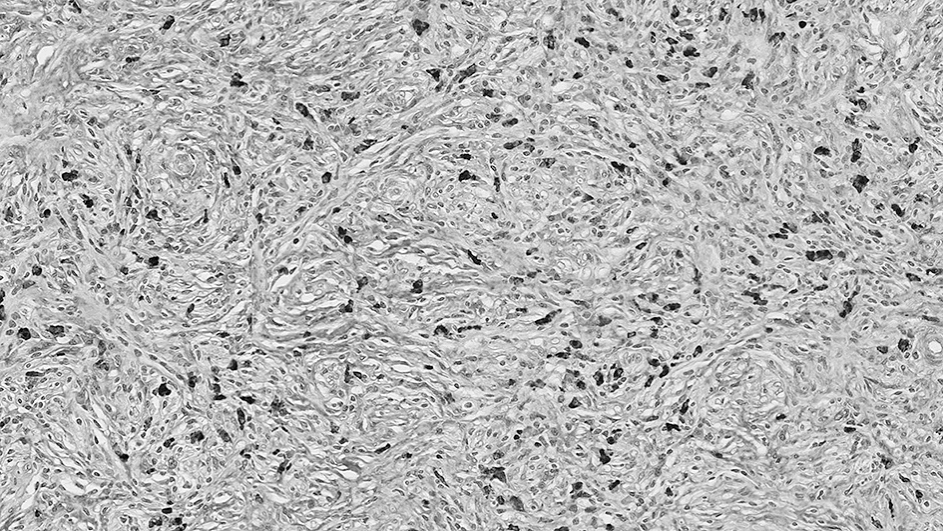
IgG4 immunostaining of pancreatic tissue in AIP showing abundant infiltration of IgG4-bearing plasma cells (×400 magnification).

In 2002, we described the characteristic histological features of abundant IgG4-bearing plasma cell infiltration in affected pancreatic tissues, which constituted another pathological hallmark of AIP (Figure 4) ^(3)^.

### (4) Systemic OOIs in AIP also responding to corticosteroid therapy

Detailed studies using computed tomography (CT) and magnetic resonance imaging (MRI) revealed that over 90% of AIP patients possessed various OOIs, including dacryoadenitis-sialadenitis (48%), bile duct lesions (78%), kidney lesions (14%), and retroperitoneal fibrosis (20%) ^(6)^. Similar to AIP, these OOIs also favorably responded to corticosteroid therapy ^(5)^.

### (5) OOIs also exhibited abundant IgG4-bearing plasma cell infiltration

In 2002, we witnessed that OOIs featured abundant IgG4-bearing plasma cell infiltration and fibrosis similar to AIP, indicating that AIP and systemic OOIs shared a common pathological background ^(3)^.

### (6) Proposal of a new systemic disease concept related to IgG4: IgG4-RD

The fact that AIP and its OOIs shared the common clinical features of abundant IgG4-bearing plasma cell infiltration and a good response to corticosteroids implied the presence of a systemic disease consisting of AIP and systemic OOIs that was closely associated with IgG4. In previous studies, experts in the field of rheumatology have revealed that Mikulicz’s disease also exhibited complicating systemic extra-glandular lesions associated with IgG4 ^(12), (13)^, and a number of nomenclatures were postulated for this systemic condition ^(13), (14), (15), (16)^. In 2010, the new disease concept of IgG4-RD was proposed by experts in Japan ^(17)^. This entity was accepted in 2011 at an international conference in Boston ^(7)^.

## Members of IgG4-RD

Recent studies have identified several new members of IgG4-RD, and representative works for these are summarized in Table 1.

**Table 1.** Representative Works in Each Member of IgG4-RD.

***IgG4-related pituitary and stalk lesions***	
Case report	Van der Vliet HJ, *Ann Intern Med*. 2004:141;896-7. Tanabe T, et al., *Int Med*. 2006:45;1243-7.
Review, diagnostic criteria	Shimatsu A, et al., *Endocr J*. 2009:56;1033-41. Leporati, P. et al., *J Clin Endocrinol Metab*. 2011:96; 1971-80. Shikuma J, et al., *Pituitary*. 2017:20;282-91.
Primary hypophysitis	Bernreuther C, et al., *Brain Pathol*. 2016/11/19
***IgG4-related hypertrophic pachymeningitis***	
Case report	Chan SK, *Am J Surg Pathol*. 2009:33;1249-52.
Review	De Virgilio A, et al., *Immunol Res*. 2017:65;386-394.
Nationwide survey	Yonekawa T, et al., *J Neurol Neurosurg Psychiatry*. 2014:85; 732-9.
***IgG4-related lacrimal and salivary gland lesions (Mikulicz’s disease, Küttner tumor)***	
History	Mikulicz J, *Semesters seines akademischen Wirkens in Wien. Stuttgart*, 1892:610-30. Küttner H, *Beitr Klin Chir*. 1896:15;815-34. Sjögren H, *Acta ophthalmol*. Suppl 1933:2;1-151. Morgan W, *Am J Pathol*. 1953:29;471-503. Suzuki S, et al., *The Ryumachi*. 1993:33;249-54 (in Japanese). Tsubota K, et al., *Invest Ophthalmol Vis Sci*. 2000:41;1666-73. Yamamoto M, et al., *Scand J Rheumatol*. 2004:33;432-433. Yamamoto M, et al., *Rheumatology (Oxford)*. 2005:44;227-34.
Proposal of new disease concept	Yamamoto M, et al., *Mod Rheumatol*. 2006:16:335-340. Masaki Y, et al., *Ann Rheum Dis*. 2008:68;1310-1315.
Pathogenesis, pathology, and clinical findings	Moriyama M, et al., *J Autoimmun*. 2014:51;81-8. Kitagawa S, et al., *Am J Surg Pathol*. 2005:29;783-91. Geyer JT, et al., *Am J Surg Pathol*. 2010:34;202-10. Takahashi H, et al., *J Autoimmun*. 2012:39;93-6. Yamamoto M, et al., *Mod Rheumatol*. 2015:25;199-204.
Diagnostic criteria	Masaki Y, et al., *J Rheumatol*. 2010:37:1380-5.
***IgG4-related ophthalmic disease***	
Review	Oshima K, et al., *Jpn J Ophthalmol*. 2012:56;380-2. Wallace ZS, et al., *Semin Arthritis Rheum*. 2014:43;806-17. Sogabe Y, et al., *Graefes Arch Clin Exp Ophthalmol*. 2014:252;531-8. Masaki Y, et al., *J Clin Exp Hematol*. 2011:51;13-20.
Survey	Japanese study group of IgG4-related ophthalmic disease. *Jpn J Ophthalmol*. 2013:57;573-9.
Diagnostic criteria	Goto H,et al., *Jpn J Ophthalmol*. 2015:59;1-7
***IgG4-related thyroid disease***	
AIP, IgG4-RD, and hypothyroidism	Komatsu K, et al.,* Dig Dis Sci*. 2005:50; 1052-7. Sah RP, et al., *Pancreas*. 2010:39;1114-6. Watanabe T, et al. *Scand J Rheumatol*. 2013:42;325-30.
Riedel’s thyroiditis and IgG4-RD	Comings DE, et al., *Ann Intern Med*.1967:66;884-92. Dahlgren M, et al., *Arthritis Care Res*. 2010:62:1312-8. Nagashima T, et al., *Rheumatol Int*. 2012:32;1851-2. Takeshima K, et al., *Endocr J*. 2015:62;725-31.
Hashimoto’s thyroiditis and IgG4-RD	Li Y, et al., *Pathol Int*. 2009:59;636-41. Li Y, et al., *J Clin Endocrinol Metab*. 2010:95;1309-17. Li Y, et al., *Mod Pathol*. 2012:25;1086-97. Kakudo K, Endocr J. 2012:59;273-81. Kawashima ST, et al., *Endocrine*. 2014:45;236-43.
Graves’ disease	Nishihara E, et al., *Thyroid*. 2013:23;1496-7. Takeshima K., et al., *Thyroid*. 2014:24;736-43.
***IgG4-related respiratory disease***	
Interstitial pneumonia	Taniguchi T, et al., *Gut*. 2004:53; 770-1. Hirano K, et al., *Intern Med J*. 2006:36;58-61.
Inflammatory pseudotumor	Zen Y, et al., *Hum Pathol*. 2005:36;710-7.
Hilar lymphoadenopathy	Saegusa H, et al., *Pancreas*. 2003:27;20-5.
Sarcoidosis and IgG4-RD	Ito T, et al., *Eur Respir J*. 2009:33;680-3. Yamamoto H, et al., *Eur Respir J*. 2011:38;1233-6.
Review	Matsui S, et al., *Respirology*. 2013:18;480-7.
Pathology and imaging findings	Zen Y, et al., *Am J Surg Pathol*. 2009:33;1886-93. Inoue D, et al., *Radiology*. 2009:251;260-70. Shrestha B, et al., *Am J Surg Pathol*. 2009:33;1450-62.
Diagnostic criteria	Matsui S, et al., *Respir Investig*. 2016:54;130-2.
***Autoimmune pancreatitis***	
History including pathology	Sarles H, et al., *Am J Dig Dis*. 1961:6;688-98. Sarles H, et al., *Bibl Gastroent*. 1965:7;75-120. Waldram R, et al., *Lancet*. 1975:1(7906);550-2. Nakano S, et al., *Digestive Disease*. 1978:23;75-9. Kawaguchi K, et al., *Hum Pathol*. 1991:22;387-95. Toki F, et al., *Endoscopy*. 1992:24;640. Yoshida K, et al., *Dig Dis Sci*. 1995:40;1561-8. Ito T, et al. *Dig Dis Sci*. 1997:42;1458-68. Ectors N, et al., *Gut*. 1997: 41;263-8. Wakabayashi T, et al., *Dig Dis Sci*. 1998:43;2415-25. Erkelens GW, et al., *Lancet*. 1999:354(9172);43-4. Horiuchi A, et al., *Gastrointest Endosc*. 2001:53;518-22. Hamano H, et al., *N Engl J Med*. 2001:344;732-8. Hamano H, et al., *Lancet*. 2002:359;1403-4. Klöppel G, et al., *Pancreas*. 2003:27;14-9. Notohara K, et al., *Am J Surg Pathol*. 2003:27;1119-27. Zamboni G, et al., *Virchows Arch*. 2004:445;552-63. Kim KP, et al., *Am J Gastroenterol*. 2004:99;1605-16. Suda K, et al., *Pancreas*. 2005:30;355-8. Sugumar A, et al., *Am J Gastroenterol*. 2009:104;2308-10.
Imaging findings	Horiuchi A, et al., *Am J Gastroenterol*. 1998:83;260-3. Irie H, et al., *Am J Roentgenol*. 1998:170;1323-7. Nakamoto Y, et al., *Clin Nucl Med*. 1999:24;778-80. Ichikawa T, et al., *Radiology*. 2001:221;107-16. Hasebe O, et al., *J Gastroenterol Imaging*. 2002:4;41-8. Horiuchi A, et al., *Gastrointest Endosc*. 2002:4;494-9. Wakabayashi T,et al., *Am J Gastroenterol*. 2003:98;2679-87. Hyodo N, et al., *J Gastroenterol*. 2003:38;1155-61. Numata K, et al., *J Ultrasound Med*. 2004:23;199-206. Farrell JJ, et al., *Gastrointest Endosc*. 2004:60;927-36. Sahani DV, et al., *Radiology*. 2004:233;345-52. Deshpande V, et al., *Am J Surg Pathol*. 2005:29;1464-71. Muraki T, et al., *Biliary Tract Pancreas*. 2005:26;711-6. Kawamoto S, et al., *Radiographics*. 2008:28;157-70. Takahashi N, et al., *Am J Roentgenol*. 2008:190;280-6. Chandan VS, et al., *Am J Surg Pathol*. 2008:32;1762-9. Ozaki Y, et al., *J Gastroenterol*. 2008:43;144-51. Fujinaga Y, et al., *Eur J Radiol*. 2009:76;228-38. Chang WI, et al., *Pancreas*. 2009:38;401-8. Kamisawa T, et al., *Abdom Imaging*. 2009:34;381-4. Shigekawa M, *J Hepatobiliary Pancreat Surg*. 2009:17;269-74. Nishino T et al., *J Gastroenterol*. 2010;45;988-96.
Nationwide and international surveys	Nishimori I, et al., *J Gastroenterol*. 2007:42;6-8. Kanno A, et al., *Pancreas*. 2012:41;835-839. Kanno A, et al. *Pancreas*. 2015:44;535-539. Hart PA, et al. *Gut*. 2013 :62;1771-1776.
Diagnostic criteria	Japan Pancreas Society, *Suizo*. 2002:17;585-7 (In Japanese). Okazaki K, et al., *J Gastroenterol*. 2006:4;626-631. Chari ST, et al., *Clin Gastroenterol Hepatol*. 2006:4;1010-1016. Kim KP, et al., *WJG*. 2006:12;2487-2496. Otsuki M, et al., *J Gastroenterol*. 2008:43;403-408. Shimosegawa T, et al., *Pancreas*. 2011:40;352-358. Shimosegawa T, et al., *Pancreas*. 2012:41;1341-2.
Guidelines and international consensus	Okazaki K, et al., *J Gastroenterol*. 2014:49;567-88. Kawa S, et al., *J Gastroenterol*. 2014:49;765-784. Kamisawa T, et al., *J Gastroenterol*. 2014:49;961-70. Okazaki K, et al., *Pancreatology*. 2017:17;1-6.
***IgG4-related sclerosing cholangitis***	
Case report and atypical primary sclerosing cholangitis	Kawaguchi K, *Hum Pathol*. 1991:22;387-95. Erkelens GW, et al., *Lancet*. 1999:354;43-4. Nakazawa T, et al., *Hepatogastroenterology*. 2001:48;625-30.
Inflammatory pseudotumor	Zen Y, et al., *Am J Surg Pathol*. 2004:28;1193-203. Zen Y, et al., *Mod Pathol*. 2007:20;884-94.
Cholangiography	Nakazawa T, et al., *Gastrointest Endosc*. 2004:60;937-44. Nakazawa T, et al., *Pancreas*. 2006:32;229. Kamisawa T, et al., *World J Gastroenterol*. 2008:14;3948-55. Nakazawa T, et al., *World J Gastroenterol*. 2013:19;7661-70.
PSC and IgG4-SC	Takikawa H, et al., *J Gastroenterol*. 1997:32;134-7. Takikawa H, et al., *Hepatol Res*. 2004:29;153-9. Nakazawa T, et al., *Pancreas*. 2005:30;20-5. Mendes FD, et al., *Am J Gastroenterol*. 2006:101;2070-5. Nishino T, et al., *J Gastroenterol*. 2007:42;550-9. Kubota K et al., *Diag Endosc*. 2011:23;10-16. Ohara H, et al., *J Gastroenterol Hepatol*. 2013:28;1247-51. Tanaka A, et al., *J Hepatobiliary Pancreat Sci*. 2014:21;43-50.
AIP and IgG4-SC	Nakazawa T, et al., *Histol Histopathol*. 2014:29;1-10.
Solitary IgG4-SC and cholangiocarcinoma	Hamano H, et al., *Gastrointest Endosc*. 2005:62;152-7. Hayashi K, *Hepatogastroenterology*. 2007:54;2146-51. Graham RP, et al., *Hum Pathol*. 2014:45;1722-9. Nakazawa T, et al., *World J Gastroenterol*. 2015:21;1334-43.
EUS and IDUS	Naitoh I, *J Gastroenterol*. 2009:44;1147-55. Nakazawa T et al., *Clin Endosc*. 2012:45;331-6.
Pathology	Uehara T, et al., *Pathol Int*. 2005:55;405-11. Zen Y, et al., *Semin Liver Dis*. 36:2016;242-56.
Diagnostic criteria	Ohara H, et al., *J Hepatobiliary Pancreat Sci*. 2012:19;536-42.
Treatment, prognosis	Ghazale et al., *Gastroenterology*. 2008:134;706-15.
***IgG4-related liver disease (IgG4 hepatopathy)***	
Case report	Gaburri PD, et al., *Am J Gastroenterol*. 2000:95;2391-4.
IgG4-hepatopathy	Umemura T, et al., *Hepatology*. 2007:46;463-71.
IgG4-related AIH	Umemura T, et al., *Gut*. 2007:56;1471-2. Umemura T, et al., *Hepatology*. 2010:52;389-90. Umemura T, et al., *J Gastroenterol*. 2011:46(Suppl 1);48-55. Ishizu Y, et al., *Hepatol Res*. 2016:46;601-606
Histopathology	Kamisawa T, et al., *J Gastroenterol*. 2003:38;982-4. Hirano K et al. *Clin Gastro Hepatol*. 2003;1:453-64.
Review	Nakanuma Y, et al., *Semin Liver Dis*. 2016:36;229-41.
***IgG4-related kidney disease***	
Case report	Takeda S, et al., *Nephrol Dial Transplant*. 2004:19;474-6. Uchiyama-Tanaka Y, et al., *Am J Kidney Dis*. 2004:43;e18-25.
Tubulointerstitial nephritis	Saeki T. *Clin Exp Nephrol*. 2007:11;168-73. Cornell LD, et al., *Am J Surg Pathol*. 2007:31;1586-97. Yoneda K, et al., *Am J Kidney Dis*. 2007:50;455-62. Saeki T. *Kidney Int*. 2010:78;1016-23. Raissian Y et al., *J Am Soc Nephrol*. 2011:22;1343-52. Nishi S, et al., *Clin Exp Nephrol*. 2011:15;810-9. Houghton DC, et al., *Mod Pathol*. 2011:24;1480-7. Kim TY, et al., *Clin Nephrol*. 2011:76;440-6. Yamaguchi Y, et al., *Hum Pathol*. 2012:43;536-49. Saeki T. *Kidney Int*. 2014:85;251-7.
Membranous glomerulonephritis	Alexander MP, et al., *Kidney Int*. 2013:83;455-62. Wada Y, et al., *Clin Kidney J*. 2013:6;486-90.
Clinical course	Mizushima I, et al., *Mod Rheumatol*. 2012:22;859-70. Saeki T. *Kidney Int*. 2013:84;826-33. Saeki T, et al., *Clin Exp Nephrol*. 2016:20;87-93. Mizushima I, et al., *Arthritis Res Ther*. 2016:36;283-90.
Immunology	Nakashima H, et al., *Clin Nephrol*. 2010:73;385-91.
Imaging findings	Takahashi N, et al., *Radiology*. 2007:242;791-801.
Diagnostic criteria	Kawano M, et al., *Clin Exp Nephrol*. 2011:15;615-26.
***IgG4-related retroperitoneal fibrosis***	
Ormond’s disease	Ormond JK, et al., *J Urol*. 1948:59;1072-9.
Multifocal fibrosclerosis	Comings DE, et al., *Ann Intern Med*. 1967:66;884-92. Clark A, et al., *Gastrointest Radiol*. 1988:13;30-2. Laitt RD, et al., *Gut*. 1992:33;1430-2.
Case report	Bartholomew LG, et al., *NEJM*. 1963:269;8-12. Hamano H, et al., *Lancet*. 2001:359:1403-4. Fukukura Y, et al., *AJR Am J Roentgenol*. 2003:181;993-5. Uchida K, et al., *Pancreas*. 2003:26;92-4. Ohtsubo K, et al., *JOP*. 2007:8;320-5.
Clinical study	Neild GH, et al., *BMC Med*. 2006:4:23. Zen Y, et al., *Am J Surg Pathol*. 2009:33;1833-9. Zen Y, et al., *Semin Diagn Pathol*. 2012:29;212-8. Chiba K, et al., *Intern Med*. 2013:52; 1545-51. Khosroshahi A et al., *Medicine*. 3013:92;82-91. Hara N, et al., *World J Gastroenterol*. 2014:20;16550-8.
***IgG4-related periaortitis/periarteritis***	
Inflammatory abdominal aortic aneurysm and abdominal aortic lesion	Kasashima S, et al., *Am J Surg Pathol*. 2008:32;197-204. Sakata N, et al., *Am J Surg Pathol*. 2008:32;553-559. Kasashima S, et al., *J Vasc Surg*. Matsumoto Y, et al., *Hum Pathol*. 2008:39;975-980. Tseng CW, et al., *Pathol Int*. 2008:58;421-6. 2009:49;1264-71. Qian Q, et al., *N Engl J Med*. 2009:361;1121-3. 3 Zen Y, et al., *Semin Diagn Pathol*. 2012:29;212-8.
Thoracic aortic lesions	Stone JH, et al., *Arthritis Rheum*. 2009:60;3139-3145. Ishida M, et al., *Pathol Int*. 2009:59;269-73. Stone JH, et al., *Arthritis Care Res*. 2010:62;316-22. Kasashima S, et al., *J Vasc Surg*. 2010:52;1587-1595. Takahashi M, et al., *Am J Med Sci*. 2011:341;166-9. Laco J et al., *Cardiovasc Pathol*. 2011:20;352-60. Agaimy A, et al., *Int J Clin Exp Pathol*. 2013:6;1713-22.
Medium-sized artery and coronary artery	Kasashima S, et al., *J Vasc Surg*. 2013:57;816-822. Matsumoto Y, et al., *Hum Pathol*. 2008:39;975-980. Ikutomi M, et al., *Cardiology*. 2011:120;22-6. Urabe Y, et al., *Circ Cardiovasc Imaging*. 2012:5;685-7. Tanigawa J, et al., *Hum Pathol*. 2012:43;1131-4. Stone JR. *Curr Opin Rheumatol*. 2011:23;88-94. Tran MN, et al., *Int J Cardiol*. 2015:201;33-4.
Clinical study	Inoue D, et al., *Radiology*. 2011 /08/02. Mizushima I, et al., *Arthritis Res Ther*. 2014:16;R156. Kasashima S, et al., *J Endovasc Ther*. 2014:21;589-97. Ozawa M, et al., *Arthritis Res Ther*. 2017:19;223.
***IgG4-related prostate disease***	
Case report	Yoshimura Y, et., *Intern Med*. 2007:45;897-901. Nishimori I, et al., *Intern Med*. 2007:46;1983-9. Hart PA, et al., *Int J Urol*. 2013:20;132-134. Bourlon MT, et al., *Case Rep Urol*. 2013;2013:295472.
Clinical study	Uehara T, et al., *Pathol Int*. 2008:58;118-25. Buijs J., et al., *Urology*. 2014:83;521-6.

### IgG4-related pituitary and stalk lesions

IgG4-related pituitary and stalk lesions exhibit the clinical findings of compressive optic neuropathy, panhypopituitarism, pituitary hypothyroidism, adrenocortical insufficiency, and syndromic inappropriate secretion of antidiuretic hormone, all of which require differentiation from primary hypophysitis ^(18)^.

### IgG4-related hypertrophic pachymeningitis

Hypertrophic pachymeningitis is a rare fibroinflammatory lesion that causes thickening of the dura in the cranium and/or spinal canal and presents as radiculomyelopathy, headache, and cranial nerve palsy. This condition was considered to be a member of multifocal idiopathic fibrosclerosis (MIF), a portion of which is equivalent to IgG4-RD ^(19)^.

### IgG4-related lacrimal and salivary gland lesions (Mikulicz’s disease, Küttner tumor)

**Figure 5.**
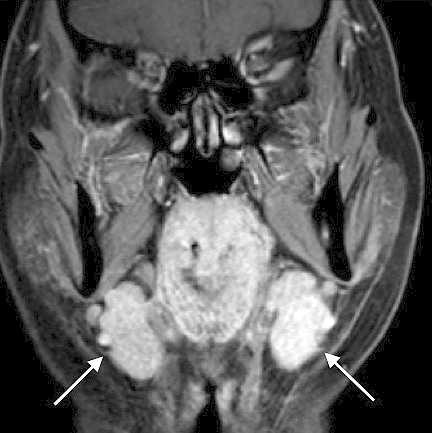
MRI findings of IgG4-related salivary gland lesions exhibiting bilateral swelling (arrows).

IgG4-related lacrimal and salivary gland lesions constitute a representative of IgG4-RDs and correspond to the existing diseases of Mikulicz’s disease and Küttner tumor. Characteristic clinical findings include symmetrical swelling of the lacrimal and salivary glands (Figure 5) with milder exocrine dysfunction and negative results for anti-SS-A/Ro and anti-SS-B/La autoantibodies, in contrast to those of Sjögren’s syndrome ^(12), (13), (16), (20)^.

### IgG4-related ophthalmic disease

Patients with IgG4-related lacrimal gland lesions sometimes develop complicating ocular adnexa lesions, such as trigeminal nerve branch enlargement, extraocular muscle enlargement, diffuse orbital fat lesions, orbital mass lesions, eyelid lesions, and nasolacrimal duct lesions, all of which might occur independently. IgG4-related ophthalmic diseases were newly proposed to integrate ocular adnexa lesions with IgG4-related lacrimal gland lesions ^(21)^.

### IgG4-related thyroid disease

AIP and IgG4-RD are significantly associated with hypothyroidism, which supports the existence of IgG4-related thyroiditis ^(22)^. Hashimoto’s thyroiditis is classified as either IgG4 or non-IgG4 thyroiditis based on immunostaining profiles ^(23)^, whereas Riedel’s thyroiditis is a chronic fibrosing disorder of an unknown etiology that is considered to be a member of MIF ^(24)^.

### IgG4-related respiratory disease

IgG4-related respiratory disease includes the imaging findings of intrathoracic lesions, such as hilar/mediastinal lymphadenopathy, bronchial wall/bronchovascular bundle thickening, interlobular septal wall thickening, nodular shadow, infiltrative shadow, pleural thickening, and effusion. IgG4-related lung disease develops through the lymphatic routes of the lungs and exhibits various clinical characteristics ^(25)^.

### Autoimmune pancreatitis

**Figure 6.**
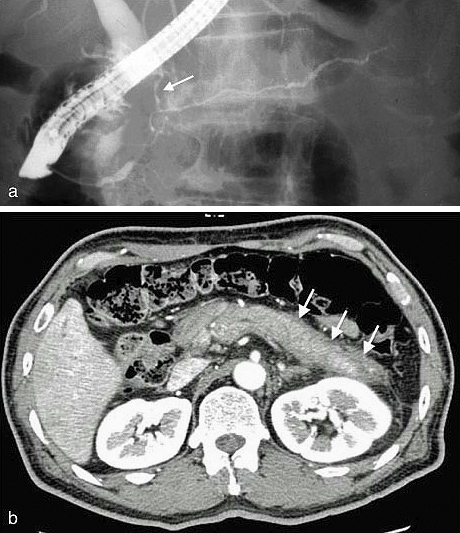
Image findings of AIP. (a) Endoscopic retrograde cholangiopancreatography (ERCP) typically shows characteristic irregular narrowing of the main pancreatic duct and narrowing of the lower bile duct (arrow). (b) Contrast-enhanced CT reveals a swollen pancreas with a straight margin and capsule-like, low-density rim (arrows). (From Shindan to Chiryo 104;453–458:2016 in a Japanese publication. Reprinted with permission from Igakutosho-shuppan, Ltd.)

AIP exhibits the typical clinical features of IgG4-RD: elderly male preponderance, high serum IgG4 levels, lymphoplasmacytic infiltration, obstructive phlebitis, storiform fibrosis, IgG4-bearing plasma cell infiltration, and a favorable response to corticosteroid therapy. Other hallmark AIP features include obstructive jaundice and pancreatic swelling, which may mimic pancreatic cancer. Several autoantibodies are positive in IgG4-RD-associated AIP, whereas other disease-specific ones, such as anti-SSA/Ro, anti-SSB/La, and anti-mitochondrial antibodies, are negative. The imaging results of irregular narrowing of the main pancreatic duct (Figure 6a) and capsule-like rim (Figure 6b) are specific and very useful for AIP diagnosis ^(1), (26)^.

### IgG4-related sclerosing cholangitis

**Figure 7.**
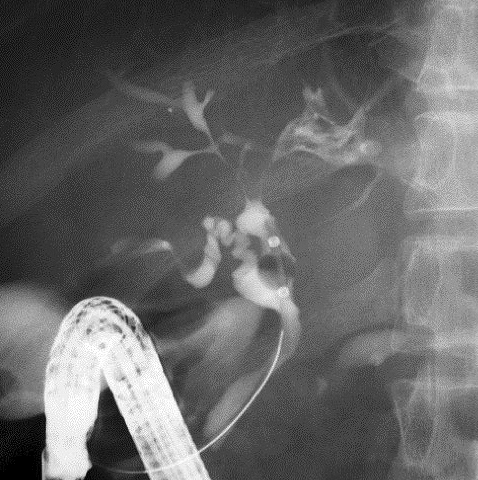
ERCP of IgG4-SC complicated with AIP showing characteristic intra-hepatic bile duct changes that are similar to those in primary sclerosing cholangitis.

IgG4-related sclerosing cholangitis (IgG4-SC) is widely distributed throughout the biliary system; therefore, lower bile duct lesions need differentiation from pancreatic cancer and intra-hepatic or hilar lesions of primary sclerosing cholangitis or biliary malignancies (Figure 7). IgG4-SC is closely associated with AIP, and their co-occurrence aids in the diagnosis of both conditions ^(27)^.

### IgG4-related liver disease (IgG4 hepatopathy)

IgG4-related liver disease includes some histological changes, all of which are collectively designated as IgG4 hepatopathy ^(28)^. Some lesions mimic those observed in autoimmune hepatitis (AIH), fulfill its diagnostic criteria, and as such are designated as IgG4-related AIH ^(29)^.

### IgG4-related kidney disease

IgG4-related kidney disease (IgG4-RKD) is characterized by uriniferous tubules displaying tubulointerstitial nephritis and sparse glomerular lesions with membranous glomerulonephritis. Most patients are diagnosed based on incidental imaging abnormalities and have only mild urinary dysfunction, whereas some develop acute or progressive renal failure ^(30), (31), (32)^.

### IgG4-related retroperitoneal fibrosis

IgG4-related retroperitoneal fibrosis is disclosed by the CT or MRI findings of soft tissue density or masses around the aorta and ureter. Peri-ureteral lesions sometimes cause symptoms of lumbago or back pain due to hydronephrosis, which may result in renal atrophy and failure ^(3), (31)^.

### IgG4-related periaortitis/periarteritis

The periaortic lesions originally found in retroperitoneal fibrosis later shows systemic distribution, which has led to the proposal of the distinct systemic disease entity of IgG4-related periaortitis/periarteritis. IgG4-related arterial lesions are characterized by arterial wall thickening mainly in the adventitia that responds well to corticosteroid treatment. However, patients with prior luminal dilatation have shown symptom exacerbation after therapy ^(33), (34), (35)^.

### IgG4-related prostate disease

Patients with IgG4-RD sometimes display ameliorated prostatic symptoms after corticosteroid treatment, suggesting the presence of IgG4-related prostate diseases that present as a symmetrically affected, nontender, swollen prostate in imaging analyses ^(36)^.

### Candidate members of IgG4-RD and uncommon manifestations of each IgG4-RD

Many other organ manifestations have been proposed as candidates of IgG4-RD, including lesions of the skin, gastrointestinal tract, mesentery, bone, and joints, mainly due to the pathological findings of IgG4-bearing plasma cell infiltration and fibrosis as well as simultaneous existence of other IgG4-RD members. In addition, each IgG4-RD may present with uncommon manifestations, many of which were reported as case reports and need further studies to confirm their association with IgG4-RD ^(37)^.

## Case of Focal AIP Complicated with IgG4-RKD and IgG4-related Periaortitis

**Figure 8.**
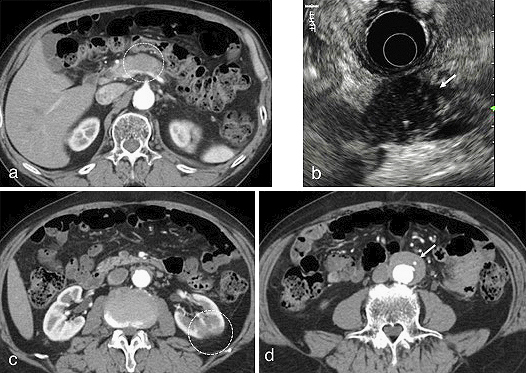
Image findings of a 72-year-old man with focal AIP. (a) Contrast-enhanced CT disclosed a localized attenuated mass at the pancreatic body (circle). (b) EUS showed a low-echo pancreatic mass with high-echo spots (arrow). (c) Contrast-enhanced CT revealed left kidney involvement with renal cortical lesions of decreased enhancement (circle). (d) Contrast-enhanced CT showed a soft tissue mass around the abdominal aorta (arrow).

A 72-year-old man was found to have a localized mass in the pancreatic body by abdominal ultrasonogram during a routine medical checkup. Since his serum IgG4 concentration was high at 412 mg/dl, localized AIP was suspected, and he was referred to Shinshu University Hospital for further examination. Contrast-enhanced CT revealed a localized attenuated mass at the pancreatic body (Figure 8a) that appeared as a low-echo mass with high-echo spots on endoscopic ultrasonography (EUS) (Figure 8b). CT also revealed left kidney involvement with renal cortical lesions of decreased enhancement (Figure 8c) and a soft tissue mass around the abdominal aorta (Figure 8d). After the exclusion malignancy by endoscopic ultrasound-guided fine needle aspiration biopsy, he was diagnosed as having focal AIP complicated with IgG4-RKD and IgG4-related periaortitis.

## Diagnosis of IgG4-RD

With IgG4-RD now being diagnosed worldwide, international collaboration has produced consensus publications on its nomenclature, pathological findings, and management approaches ^(38), (39), (40)^. The diagnostic criteria of IgG4-RD used in the routine clinical care of patients are generally heterogeneous and reflect the various features of the disease to identify as many people with condition as possible ^(41)^. The comprehensive diagnostic criteria for IgG4-RD were at first intended by Japanese researchers to be used by general physicians and nonspecialists ^(42)^. Although IgG4-RD manifestations in several organs exhibit clinical and pathological differences, a consensus has been reached on two points for the definite diagnosis of IgG4-RD: (i) serum IgG4 levels >135 mg/dl and (ii) >40% of IgG+ plasma cells being IgG4+, with >10 IgG4+ cells per high-power field of a biopsied sample. These criteria also require precise differentiation from malignant tumors and mimicking conditions using additional histopathological examination. However, it can be difficult to obtain tissue samples from certain organs, such as the pancreas; organ-specific diagnostic criteria including those for AIP ^(43)^, IgG4-related sialadenitis and dacryoadenitis ^(44)^, IgG4-RKD ^(45)^, IgG4-SC ^(46)^, and IgG4-related respiratory disease ^(25)^ are therefore available for such lesions in the form of diagnostic algorithms. The framework of these organ-specific criteria is largely based on that of AIP to include combinations of the following: (i) characteristic imaging findings, (ii) elevation of serum IgG4 levels, (iii) histopathological findings of abundant IgG4-positive plasma cell and lymphocyte infiltration, storiform fibrosis, and/or obliterative phlebitis, (iv) association with other IgG4-RDs, and (v) a favorable response to steroids. The algorithms involved in this diagnostic process have remarkably increased detection sensitivity.

Classification criteria are standardized definitions that are primarily intended to create well-defined, relatively homogeneous cohorts for clinical research. They are not intended to capture the entire universe of possible patients but rather to capture the majority of patients with key shared features of the condition ^(41)^. Classification criteria for IgG4-RD to differentiate between IgG4-RD and mimickers are needed.

## Treatment of IgG4-RD

Oral glucocorticoids are the first-line agents for remission induction in patients with active IgG4-RD or in whom vital organs are involved or serious organ dysfunction or failure is likely ^(40), (47), (48), (49)^. Not all forms of IgG4-RD require immediate treatment, and simple observation is sufficient in some cases ^(7)^. Following a successful course of induction therapy, patients with high disease activity may benefit from maintenance therapy ^(50)^. Although several immunomodulatory agents have been adopted as steroid-sparing agents for IgG4-RD, only little evidence is available supporting the effectiveness of these drugs ^(51)^. B-cell depletion with Rituximab (RTX) has been effective for treating IgG4-RD, even for patients refractory to conventional steroid-sparing agents; high complete remission rates following RTX therapy were reported in groups of patients whose disease had been resistant to, or who had contraindications for, steroids or conventional steroid-sparing agents ^(40), (51)^. Moreover, the number of plasmablasts that were largely IgG4-positive and played a role in disease activity sharply decreased after RTX treatment, presumably due to depletion of CD20^+^ precursors ^(52)^.

The IgG4-RD Responder Index (RI) was developed to help investigators assess treatment efficacy in a structured manner. RI guides examiners through assessments of disease activity and damage, incorporating higher weights for disease manifestations that require urgent treatment or worsen despite therapy. An international multispecialty study has confirmed RI as a valid and reliable disease activity assessment tool to measure treatment response ^(53)^.

## Long-term Prognosis of IgG4-RD

IgG4-RD had originally been considered an acute-phase condition due to its favorable response to corticosteroids and pathological findings of lymphoplasmacytic infiltration, both of which suggest a reversible condition with a good prognosis. However, recent studies have found that long-term disease affliction may instead transition to (1) advanced-stage conditions with dysfunction or (2) complicating malignancy.

### (1) Transition to advanced-stage conditions exhibiting dysfunction

**Figure 9.**
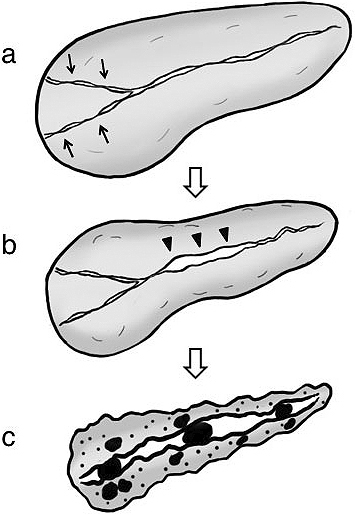
Sequential progression mechanism of AIP to confirmed chronic pancreatitis. (a) Narrowing of both Wirsung’s and Santorini’s ducts (arrows) by pancreatic head swelling causes pancreatic juice stasis in the upstream pancreatic duct. (b) Pancreatic juice stasis results in increased intrapancreatic duct pressure that is resistant to typical AIP-specific main pancreatic duct narrowing in the pancreatic body region, leading to ductal nonnarrowing in this region (arrowheads). (c) In concert with relapse, these events ultimately result in severe calcification. (From Ref. 51. Reprinted with permission.)

Some AIP cases showed pancreatic calcification and atrophy during the long-term course, which were the hallmarks of chronic pancreatitis with functional abnormality. Based on cumulative imaging and functional study outcomes, we proposed a sequential progression mechanism of AIP to chronic pancreatitis, whereby pancreatic head swelling during the acute stage leads to long-standing narrowing of pancreatic ducts in the head region that may then cause pancreatic juice stasis in the upstream pancreatic duct. In concert with multiple recurrences, these events can severely damage pancreatic tissues and ultimately result in severe calcification of the entire pancreas and pancreatic atrophy (Figure 9) ^(54)^. Since frequent recurrences had a significant impact on the progression to a chronic stage, measures to prevent them might improve long-term prognosis.

In a previous study, IgG4-related dacryoadenitis and sialadenitis have also been reported to recur over time; delayed treatment-induced fibrosis progression resulted in exocrine dysfunction due to diminished salivary secretion ^(20)^. IgG4-RKD responded well to corticosteroid therapy in the long-term course, but eventual renal atrophy was associated with lower pretreatment estimated glomerular filtration rate, suggesting a risk of chronic stage progession ^(32)^. Finally, despite IgG4-related periaortitis generally responding favorably to corticosteroid treatment, some cases of prior luminal dilatation showed disease exacerbation after therapy ^(35)^.

In summary, IgG4-RDs tend to respond well to corticosteroid therapy over the long-term course and exhibit a good outcome. However, progression to a chronic, dysfunctional state appears to be closely related to the number of recurrences and/or degree of pretreatment or early functional impairment.

### (2) Complicating malignancy

A close association of IgG4-RD with malignancy in the elderly has been reported by Yamamoto et al. ^(55)^ and Shiokawa et al.^(56)^ that was presumably due to persistent inflammatory tissue damage or impaired immunosurveilance. However, other studies including those by Hirano et al. ^(57)^ and Hart et al.^(58)^ have contradicted these relationships because most of the cases of IgG4-RD were based on imaging tests in large-volume centers that included patients more likely to be diagnosed with malignancies, especially during the first year after diagnosis. Indeed, we also identified a close association between IgG4-RD and malignancy within 12 years after diagnosis, particularly during the first year and mostly during the first 5 years. An active IgG4-RD state is suspected to be a strong risk factor for malignancy development ^(59)^. Further studies are needed to clarify this notion.

## Immunological Abnormalities in IgG4-RD

The immune system is broadly divided into innate and adaptive components. The innate immune system functions immediately after exposure to pathogens, followed by adaptive immune function as a result of clonal expansion. The main distinction of these systems lies in the mechanisms and receptors used for immune recognition. In the adaptive immune system, an extremely diverse repertoire of receptors is randomly generated, and lymphocytes bearing specific receptors are subsequently selected for clonal expansion after encountering specific antigens. Such receptors cannot be genetically passed to the next generation. In contrast, innate immune recognition is mediated by germ line-encoded receptors, meaning that the specificity of each receptor is genetically predetermined. The strategy of the innate immune response may not be to recognize every possible antigen but rather to focus on several highly conserved structures, i.e., pathogen-associated molecular patterns (PAMPs) that are present in large groups of microorganisms ^(60)^. The immunological abnormalities in IgG4-RD have been related to both (1) adaptive and (2) innate immune systems.

### (1) The adaptive immune system

IgG4-RD is associated with Th2-type immune balance and abundant infiltration of regulatory T-cells (Tregs) into target organs. The cytokine profile of IgG4-RD includes Th2 cytokines (IL-4, IL-5, and IL-13) as well as regulatory cytokines (IL-10 and TGF-β) ^(61), (62), (63)^. Increased peripheral Tregs are positively correlated with serum IgG4 levels. Inducible costimulator (ICOS)-positive Tregs may influence IgG4 production via IL-10, whereas ICOS-negative Tregs may modulate fibrosis via TGF-β ^(63), (64)^.

The pathological effects of IgG4 and other autoantibodies have been reported to evoke IgG4-RD. Shiokawa et al. ^(65)^ demonstrated that subcutaneous injection of IgG1 or IgG4 from AIP patients, but not control IgGs, caused pancreatic and salivary gland injury in neonatal mice. Since the potent pathogenic activity of IgG1 was significantly inhibited by simultaneous IgG4 injections, circulating IgG4 in AIP patients was considered to exert both pathogenic and protective roles. Moreover, the autoantibodies in AIP serum reacted to molecules involved in cell-extracellular matrix adhesion, which could explain the systemic distribution of this disease ^(65)^. Their recent study identified autoantibodies against laminin 511-E8 in many AIP patients and autoantibodies against integrin α6β1, a ligand for laminin 511, in a limited number of patients, and immunization with laminin 511-E8 induced histological changes of acinar atrophy, fibrosis, dense infiltration of plasma cells and lymphocytes, and obliterative phlebitis in the pancreas of mice. These autoantibodies may exert pathogenic activities by targeting laminin 511/integrin α6β1 binding and provide a good marker for the diagnosis and therapeutic effects ^(66)^.

Molecular mimicry by either a virus or bacteria has also been hypothesized to initiate or exacerbate autoimmune responses through sequence or structural similarities with self-antigens. *Helicobacter pylori* infection may trigger AIP, possibly as a result of the substantial homology between human carbonic anhydrase (CA) II and *H. pylori* alpha-CA. The homologous segments contain the binding motif of the HLA molecule DRB1*04:05, which is closely associated with AIP ^(67)^. The HLA-DRB1*04:05 allele was also an important risk factor for AIP in functional studies using a transgenic mouse model ^(68)^. These data indicated that the DRB1*0405-restricted peptide of CA II was present in genetically predisposed subjects, which resulted in reactive T-cells injuring pancreatic tissue via interactions with the CA II of pancreatic ductal cells ^(69)^.

### (2) The innate immune system

Functionally, the pattern-recognition receptors of the innate immune system can be divided into three classes: secreted, endocytotic, and signaling. Mannan-binding lectin is a secreted pattern-recognition receptor that binds to microbial carbohydrates to initiate the lectin pathway of complement activation ^(60)^. The complement activation system may play a role in the pathogenesis of IgG4-RD, since decreased serum levels of complement proteins are sometimes found in IgG4-RD, especially in IgG4-RKD. Sugimoto et al. ^(70)^ observed that both the classical and mannose-binding lectin pathways might participate in the complement activation system of IgG4-RD. However, we uncovered significantly higher serum IgG1 levels and decreased C3 and C4 values in an elevated immune complex group, which indicated that IgG1, and not IgG4, contributed to complement activation via the classical pathway ^(71)^.

Basophils seem to be the key players in the innate immune system of IgG4-RD via their signaling pattern-recognition molecules of toll-like receptors (TLRs) and nucleotide-binding oligomerization domain-like receptors (NDRs) ^(72), (73)^. Activated basophils enhance IgG4 production in B-cells by B-cell-activating factor, leading to the differentiation of inflammatory monocytes into M2 macrophages that affect the Th2 immune environment and promote fibrosis through secretion of TGF-β. TLRs recognize PAMPs and damage-associated molecular patterns (DAMPs). PAMPs derived from abundant gut bacteria or DAMPs from necrotic cells in damaged organs may induce tissue injury and inflammation via their TLR or NDR signaling pattern-recognition molecules ^(63)^.

Uchida and Okazaki ^(63)^ proposed a hypothesis and a diagram for the pathogenesis of type 1 AIP based on their studies as follows: In the initial stage of type 1 AIP, because of the decreased naïve Tregs and CD19^+^ CD24^high^ CD27^+^ Bregs, effector T-cells are involved in tissue damage. IL-10 and TGF-β from increased inducible Tregs induce the switch from B-cells to IgG4-producing plasma cells and fibrosis, respectively. Basophils lead to differentiation of inflammatory monocytes into M2 macrophages, affect production of IgG4 via TLR signaling, and influence the Th2 immune environment. M2 macrophages also contribute to fibrosis and Th2 immune reaction ^(63)^.

Animal models of AIP have been developed by exposing C57BL/6 mice to heat-killed *Escherichia coli* to produce marked cellular infiltration with fibrosis in the exocrine pancreas and salivary glands ^(74)^. One representative antigen reacting with the serum of *E. coli*-inoculated mice was FliC ^(75)^. These findings indicate that avirulent bacteria and other silently infiltrating microorganisms can engage PAMPs and activate the innate immune system to elicit a host immune response to the target antigen by molecular mimicry, which can ultimately lead to AIP.

## Immunogenetic Background of IgG4-RD

The pathogenesis of autoimmune diseases is multifactorial and involves the complex interplay of multiple genetic and environmental factors. To elucidate the genetic elements that influence disease susceptibility, two approaches have been employed: (1) the candidate-gene approach by association studies and (2) genome-wide association study (GWAS) of large numbers of single-nucleotide polymorphisms ^(76)^.

### (1) Association studies using polymorphic markers in candidate genes

Among the major histocompatibility complex class I and II molecules, the HLA serotypes of DR4 and DQ4 are the markers most frequently associated with AIP. Specifically, the frequencies of DRB1*0405 and DQB1*0401 are significantly higher in AIP patients. In the Japanese population, DRB1*0405 is known to be in strong linkage disequilibrium with DQB1*0401, resulting in the DRB1*0405-DQB1*0401 haplotype ^(67)^. We have identified several other candidate genes involved in the pathogenesis of AIP, including Fc receptor-like 3 (*FCRL3*)**and the cytotoxic T lymphocyte antigen 4**(*CTLA4*) ^(76)^.

### (2) Association studies using genome-wide polymorphic markers

A large association analysis of AIP using microsatellite markers revealed relationships with potassium voltage-gated channel, shaker-related subfamily, member 3 (*KCNA3*). *KCNA3* is involved in the immunomodulation of autoreactive effector T-cell- and memory T-cell-mediated autoimmune diseases ^(77)^. GWAS results have also identified candidate genes in the development of lachrymal/salivary gland lesions complicated with AIP ^(76)^.

## Precise Role of IgG4 in the Pathogenesis of IgG4-RD

Although IgG4 has a key role in the diagnosis of IgG4-RD, it is unclear whether it also has a pathogenic role or a protective one or if it is merely an innocent bystander. The pathogenic function of IgG4-type autoantibodies on self-antigens caused injury in limited settings, such as in pemphigus vulgaris, idiopathic membranous glomerulonephritis, acquired thrombotic thrombocytopenic purpura, and muscle-specific kinase myasthenia gravis. In agreement with this, Shiokawa et al. ^(65)^ reported that IgG4 from AIP patients caused pancreatic and salivary gland injuries in neonatal mice but also attenuated the injurious effects of injected IgG1.

**Figure 10.**
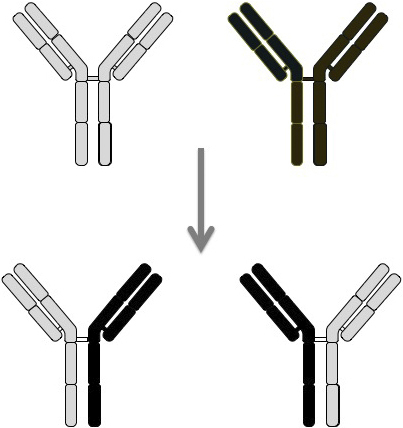
Fab-arm exchange, a characteristic feature of IgG4.

In contrast to the other IgG subclasses, IgG4 lacks the conventional IgG activity of complement-Fcγ receptor binding and has a relatively low serum concentration, both of which suggest that IgG4 has regulatory rather than inflammatory or antigen-clearance functions ^(78)^. This may be, in part, due to the inherent instability of IgG4, whereby the interchain disulfide bonds in the hinge region are labile, thus allowing the molecule to split and exchange monovalent halves, called Fab-arm exchange (Figure 10) ^(79)^. It has been estimated that up to half of IgG4 molecules may be dissociated, existing as single heavy-chain and light-chain molecules in the serum. Such weak antigen binding of IgG4 monomers or bivalent Fab molecules could prevent the formation of immune complexes of other IgG subclasses ^(78)^. Although IgG4 bound to IgG exerted rheumatoid factor activity, IgG4 binding to IgG Fc occurred via Fc–Fc interactions and not rheumatoid activity per se ^(80)^. Different IgG4 molecules with the same antigen reactivity may associate through Fc–Fc interactions and then exchange molecule halves, resulting in Fab-arm exchange with different epitope reactivity.

## What Is Needed to Understand the Entire Picture of IgG4-RD?

The discovery of a close association between AIP and IgG4 has opened the door to the new disease concept of IgG4-RD, which shows the unique clinical features never encountered previously. In order to understand the whole picture of IgG4-RD, researchers need to clarify the precise spectrum of IgG4-RD and real global prevalence, rational treatment strategies, long-term outcomes, pathogenesis, and finally the exact role of IgG4. Many individuals with IgG4-RD worldwide have been overlooked or misdiagnosed. Expanding the global awareness of IgG4-RD will result in enhanced diagnosis, improved treatment, and the scope for larger international studies in the future.

## Article Information

### Conflicts of Interest

None

### Sources of Funding

This work was partially supported by a Grant-in-Aid for ‘‘Research for Intractable Diseases’’ Program from the Ministry of Labor and Welfare of Japan, grant number [H29-Nanchitou-(Nan)-Ippan-058].

### Acknowledgement

We thank Dr. Takayuki Watanabe and Dr. Yasuhiro Kuraishi for their assistance in figures preparation and Mr. Trevor Ralph for his English editorial assistance.

### Author Contributions

- Substantial contributions to the conception or design of the work; or the acquisition, analysis, or interpretation of data for the work; AND
- Drafting the work or revising it critically for important intellectual content; AND
- Final approval of the version to be published; AND
- Agreement to be accountable for all aspects of the work in ensuring that questions related to the accuracy or integrity of any part of the work are appropriately investigated and resolved.

### Approval code issued by the institutional review board (IRB) and the name of the institution(s) that granted the approval

This manuscript is a review article and does not need approval by IRB.
